# Active Optical Fibers and Components for Fiber Lasers Emitting in the 2-μm Spectral Range

**DOI:** 10.3390/ma13225177

**Published:** 2020-11-17

**Authors:** Filip Todorov, Jan Aubrecht, Pavel Peterka, Ondřej Schreiber, Ali A. Jasim, Jan Mrázek, Ondřej Podrazký, Michal Kamrádek, Nithyanandan Kanagaraj, Martin Grábner, Yauhen Baravets, Jakub Cajzl, Pavel Koška, Adam Fišar, Ivan Kašík, Pavel Honzátko

**Affiliations:** 1 Institute of Photonics and Electronics of the Czech Academy of Sciences, Chaberská 57, 182 51 Prague, Czech Republic; aubrecht@ufe.cz (J.A.); peterka@ufe.cz (P.P.); schreiber@ufe.cz (O.S.); jasim@ufe.cz (A.A.J.); mrazek@ufe.cz (J.M.); podrazky@ufe.cz (O.P.); kamradek@ufe.cz (M.K.); nithi.physics@gmail.com (N.K.); grabner@ufe.cz (M.G.); baravets@ufe.cz (Y.B.); cajzl@ufe.cz (J.C.); its@ufe.cz (P.K.); kasik@ufe.cz (I.K.); honzatko@ufe.cz (P.H.); 2 Faculty of Nuclear Sciences and Physical Engineering, Czech Technical University in Prague, Břehová 7, 115 19 Prague, Czech Republic; 3 Optoelectronic Research Center, University of Southampton, Southampton, Hampshire SO17 1BJ, UK; 4 SQS Vláknová Optika, a.s., 509 01 Nova Paka, Czech Republic; adam.fisar@sqs-fiber.cz

**Keywords:** rare-earth (RE) doped optical fibers, nanoparticle doping, fiber lasers, preform shaping, fused fiber components, Bragg gratings, mode-locked fiber lasers, self-swept fiber lasers, fiber laser material processing

## Abstract

Laser sources emitting in the infrared range at around 2 µm are attracting great interest for a variety of applications like processing of transparent thermoplastic polymers in industry as well as plenty of applications in medicine, spectroscopy, gas sensing, nonlinear frequency conversion to the mid-infrared, to mention a few. Of late, fiber lasers compared to other kinds of lasers benefit from their all-fiber design, leading to a compact, robust, and well thermally manageable device. Particularly, thulium- and holmium-doped fiber lasers are the first choice in fiber lasers emitting light around 2 µm. In this paper, we give an overview of our recent results in the research on thulium- and holmium-doped optical fibers, fiber lasers, and related research topics in the 2-µm spectral range. In particular, we present, to our knowledge, the first results of improvement of pump absorption in double-clad fibers thanks to the fiber twist frozen during drawing. Finally, a brief demonstration of material processing by thulium all-fiber laser operating at 2 µm is presented.

## 1. Introduction

Many processes in industry, sensing, defense, medicine, and other branches of human activities could accommodate lasers with all-fiber configuration working in the 2-μm wavelength range. Particularly, thulium-doped (Tm), holmium-doped (Ho), or thulium and holmium co-doped (Tm:Ho) fiber lasers have been demonstrated [1]. They belong to the group of so-called eye-safe lasers since the light with a wavelength longer than 1.4 μm is strongly absorbed in the human eye’s vitreous parts and therefore it is generally less probable to damage the retina [2]. This fact, with respect to the rest of the parameters of the laser sources (e.g., optical intensity, beam divergence, and quality), allows a potentially lower safety and security demand for the laser workplace and enables their usage in free-space optical communication systems or in LIDAR (Light Detection And Ranging) and gas sensing applications [3,4]. High-power Tm fiber lasers could be employed in material processing applications [5,6]. Owing to the efficient light absorption at the 2-μm wavelength range, processing of transparent thermoplastics by Tm laser is possible without the usage of any additional additives in the processed polymers, which is a significant benefit compared to the requirements of the 1-μm ytterbium laser processing technology widely used nowadays [7]. This feature of Tm lasers expands the processing options with a wider array of materials. Either thermal ablation by using CW lasers or cold ablation by using ultrafast pulsed lasers (minimizing the material’s thermal load) are involved in material processing applications [8]. Certain medical applications like lithotripsy (fragmentation of kidney stones) or prostate enucleation (treatment of benign prostatic hyperplasia) could benefit from the working parameters of Tm and Ho fiber lasers as well [9,10,11]. Particularly, important applications of lasers emitting at 2 µm are pumping of solid state lasers (crystals, Ho-doped fiber) or in nonlinear frequency conversion to mid-infrared wavelength for spectroscopy and generation of supercontinuum [12,13].

**Figure 1:**
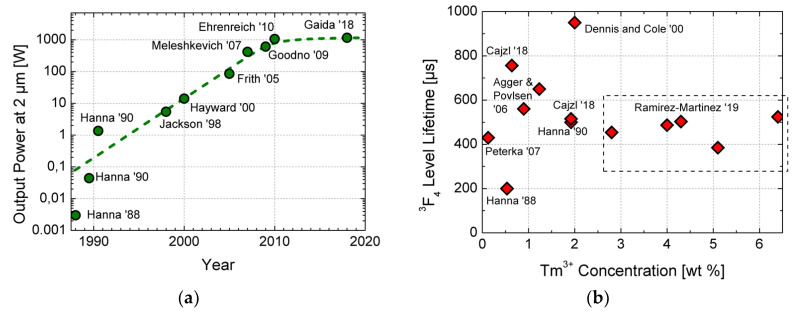
Power evolution with time in Tm-doped fiber lasers (**a**) and one of the key spectroscopic characteristics of Tm-doped fibers, the lifetime of the upper laser level ^3^F_4_ vs. thulium concentration (**b**). Selected results are presented in both schematic figures, the respective reference numbers [14,15,18,19,20,21,22,23,24,25,26,27,28,29,30] are shown next to the graph points.

Rare earth (RE) thulium- and holmium-doped fiber lasers emitting light at around 2 µm could reach high powers (kW-levels for Tm were demonstrated for a short timescale in laboratory conditions [14,15]) and high slope efficiency whilst having relaxed non-linear limits and they are already challenging the well-established ytterbium-doped fiber lasers operating at around 1 µm [16,17]. Selected research results showing laser output power evolution of thulium fiber laser are reviewed in Figure 1a: from the pioneering results at the end of eighties [18,19,20] over the start of exploitation of the so-called two-for-one cross-relaxation process for pumping [21,22,23], pumping by high-power erbium fiber lasers [24] at the turn of millennium that culminated with about 1 kW output power [14,15,25]. Most efficient, high power thulium fiber laser experiments required a high concentration of thulium over 3.5 wt %. The exception is the record experiment [14] where the thulium concentration of about 1 wt % was reported but without further details about the homogeneity of dopants over the large core. Currently, the main power limiting factor refers to heat management; it can be mitigated to some extent by optimization and tailoring of pump absorption that is one of the topics addressed in this paper. One of the key spectroscopic parameter of thulium fibers is the lifetime of the thulium energy level ^3^F_4_ that is the metastable level for the thulium lasers at around 2 µm.

Unlike the output power, the lifetime is not critically dependent on thulium concentration, but rather it depends on the fabrication process and Tm^3+^ local environment in the host material. Some representative reported values are schematically plotted in Figure 1b [18,19,20,26,27,28,29,30]. The record value of almost 1 ms was reported by Michael Dennis and Brian Cole in silica fiber highly doped with GeO_2_ and Al_2_O_3_ [30]. Improvement of the ^3^F_4_ lifetime can be achieved also by the nanoparticle doping method [26] that is briefly reviewed in the following section.

Being a growing technology, the development of 2-µm fiber lasers has been hindered by several factors, for instance, the availability of specialty optical fibers, fiber components (couplers and splitters, wave-division multiplexers, isolators), and suitable high-power pumping laser diodes on the market or by other issues, e.g., by thermal performance of the fiber polymer coatings. There were several works published of late to address or to overcome some of the limitations, e.g., Tm:Ho co-doping of silica fibers [31] or pumping the Ho-doped fiber laser by a Tm-doped one [32]. Nevertheless, there is still a lot of room for further developments.

In this paper, we review our recent progress in the research of Tm- and Ho-doped optical fibers, fiber components, fiber lasers, and broadband light sources working at the 2-µm spectral range. Results of our research on the increasing of the pump absorption of double-clad fibers, fiber laser self-sweeping, fiber lasers for material processing, and other related topics are briefly described as well.

## 2. Processing Methods and Composition of Active Optical Fibers

Despite the progress in the field of single crystal fibers [33] and transparent ceramics [34], the RE-doped glasses play the key role for the preparation of active optical fibers, and thus remain at the heart of amplifier and laser technology. Special attention has been paid to the silica glass fibers for their high thermal stability and durability which plays an important role in the construction of high-power lasers. However, the luminescence efficiency of RE-doped fibers is strongly limited by the physicochemical properties of the host glass matrix. The low solubility of RE and the high phonon energy of silica glass can be considered as the two major factors the luminescence efficiency depends on. Once the concentration of RE ions in silica glass matrix exceeds the solubility limits; that are lower than several tens of ppm [35]; phase separation takes place and the RE ions form clusters. The inter-ionic non-radiative energy transfers are preferred over the radiative decays in the RE clusters causing significant decreases of luminescence efficiency [36]. The high phonon energy contributes to non-radiative energy transfers between particular RE manifolds [37] as a competitive process to the radiative decay. Both phenomena are especially remarkable in Tm-doped fiber laser devices for the 2-μm laser transition where they have relatively high non-radiative decay rates and the achievable quantum conversion efficiency falls to about 10% only. That is in contrast to almost 100% efficiencies at 1 μm for the ytterbium- and at 1.5 μm for the erbium-doped fiber laser devices, respectively. An increased Tm quantum conversion efficiency by modification of the local environment of Tm ions by a high alumina nanoparticle co-doping is one of the efficient approaches and it is presented in ref. [38].

Ever since the seminal work of Poole et al. [39] on the general concept of RE-doped fibers, the material research has focused on novel processing methods allowing to increase the solubility limits of RE and thereby improving the luminescence efficiency of the host matrix.

Although the modified chemical vapor deposition (MCVD) technique became the technology of choice to prepare low-loss passive optical fibers [40], however, it is limited to the chemical composition that can be prepared from the corresponding gases, volatile compounds, or saturated vapors [35]. On the other hand, the solution-doping (SD) method was the first alternate technique eliminating the underlined crucial drawback [35,41,42]. In this method, the corresponding salts are dissolved and formed solutions are soaked inside the porous silica layer during the MCVD process. Needless to say, the SD method had been a leading technology to fabricate active optical fibers for more than a decade. In 2007, we have proved in ref. [43], a novel concept of nanoparticle doping (ND), where we used the colloidal solutions of ceramic nanoparticles instead of true solutions applied in the SD method. The nanoparticles play several roles and the local changes of the physicochemical properties of the host glass matrix being the most important one. The RE ions are preferentially dissolved inside the nanoparticles with lower phonon energy than a pure silica glass causing the improvement of the luminescence efficiency and increasing of the solubility limit. Lower electrostatic forces on the nanoparticles’ surface allows to increase the final dopant concentration and to also improve the spatial homogeneity of the fibers [44,45,46]. Despite the rapid development of advanced deposition methods, such as direct nanoparticle deposition [47], aerosol deposition [48], or chelate deposition [49], the ND method remained the favorable technology for its feasibility and cost effective technological equipment.

The flexibility can be considered as another great advantage of the ND method. The modification of the processing parameters of RE-doped Al_2_O_3_ nanoparticles provided a set of active fibers with tailored properties. In the case of Ho-doped fibers, a lifetime of ^5^I_8_ → ^5^I_7_ transition was longer than 1300 μs and these fibers were used in the fiber laser with slope efficiency higher than 86% of the theoretical maximum [36]. Tm-doped samples exhibiting a lifetime of ^3^F_4_ ↔ ^3^H_6_ transition longer than 570 μs and a laser slope efficiency higher than 55% with in-band pumping at around 1.6 μm were presented in ref. [50]. Modification of the chemical composition of the nanoparticles can be used to tailor the luminescence properties of the incorporated RE. For instance, silica glass doped with ZrO_2_ nanoparticles represents an interesting low phonon energy composition that was already successfully applied for a preparation of Er-doped fibers [51]. The fibers containing Tm-doped ZrO_2_ nanoparticles exhibited a two component lifetime of ^3^F_4_ ↔ ^3^H_6_ transition with a lifetime of about 680 μs and a slope efficiency of 87% of the theoretical maximum [52]. Recent research has focused on the novel low-phonon energy nanocrystalline materials [37]. Beside the application of nanocrystalline yttrium aluminum garnets [53], special attention has been paid to RE-doped pyrochlores. This class of materials exhibits very effective luminescence properties and the lifetime of ^5^I_8_ → ^5^I_7_ transition recorded for Ho-doped samples of (RE_0.05_Y_0.95_)_2_Ti_2_O_7_ was longer than 4000 μs [54]. Prepared nanocrystals with tailored structural properties were successfully incorporated in silica glass fibers [55] that are further investigated.

## 3. Unconventional Fiber Geometry and Layouts for Improved Fiber Laser Performance

Fiber lasers operating at high-power levels are typically based on the cladding-pumping principle [56,57,58]. The pump light is coupled into the inner cladding and interacts with the doped RE ions in the fiber core. The inner cladding of the double-clad (DC) fiber has a much bigger diameter than the narrow, typically single-transverse-mode core, which allows to couple light from low beam quality light sources like diode bars (high-power light sources with low price per Watt). On one hand, getting bigger inner cladding diameter and numerical aperture allows to couple more light into the fiber. But on the other hand, it means only less modes can interact with the fiber core and thus the pump absorption efficiency is generally poor. With enhanced pump absorption efficiency, the DC fiber of shorter length can be used in the fiber devices, and in such a way, the unwanted effects of background losses and nonlinear effects can be mitigated.

One of the efficient techniques to enhance the pump absorption in DC fibers is to prepare fibers with a broken circular symmetry cross section like D-shaped, hexagon, stadium, stress-element inclusion (e.g., panda-type polarization maintaining fibers), spiral cladding, etc. Breaking the fiber’s circular symmetry leads to non-regular or even chaotic paths of the propagating modes (mode scrambling), resulting in more interactions with the fiber core. This makes the excitation of the RE ions situated in the fiber core more effective and thus enhances the pump absorption.

**Figure 2:**
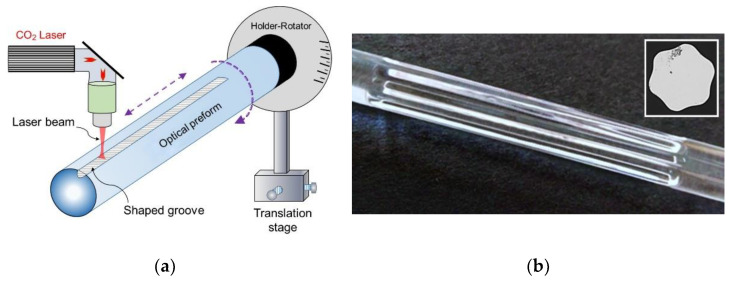
(**a**) Schematic diagram of the CO_2_ laser shaping setup; (**b**) Longitudinal section of the thermally-shaped preform with hexagonal shape (inlet: cross section).

Efforts for reaching the most effective pumping absorption often leads to a demand of inner cladding cross section geometries which are complicated to be produced by conventional mechanical grinding of the optical preform surface. To overcome this complication, a more versatile fabrication technique of CO_2_ laser thermal-based shaping, schematically shown in Figure 2, could be used [59,60]. Experimental comparison of both preform-shaping techniques was published in [61]. The mechanical shaping retains advantages for the significant reduction of the absorption peaks related to the OH content by removing the surface layer precipitated by water in the course of preform collapse in the hydrogen-oxygen flame. In thermal-shaping, the OH-groups diffuse deeper towards the preform center.

**Figure 3:**
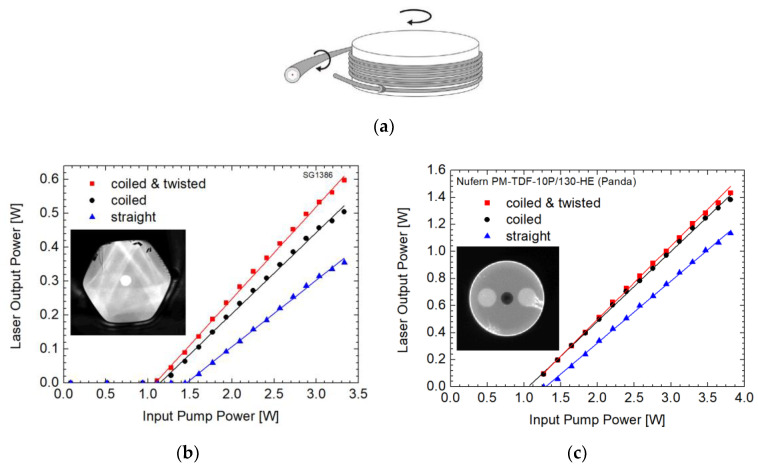
(**a**) Scheme of simultaneous coiling and twisting of the fiber; Laser characteristics under the conditions of straight, coiled, and coiled and twisted: (**b**) fiber with hexagonal shape of the inner cladding; (**c**) Panda fiber.

It is worth mentioning that the changes in the geometry along the fiber length also affect the mode scrambling. Therefore, special coiling methods like figure-eight, kidney, or spiral-shaped coiling were demonstrated for this purpose to improve the pump absorption. We developed a software tool to describe the pump absorption in coiled and twisted DC fibers [62,63]. Following that, we demonstrated (Figure 3) that simultaneous coiling and twisting either of DC fibers with hexagonal shape of the inner cladding or panda fibers may lead to substantial increase of the pump absorption along the fiber. Correspondingly, it may lead also to the increase of the fiber laser slope efficiency for the given active fiber length. The coiling and twisting was applied to the drawn fiber and the effect on the pump absorption was discussed in ref. [64,65]. Note that the hexagonal fiber was cut intentionally much shorter than the estimated optimal fiber length for getting absorbed at least 95% (13 dB) of the pump. Under standard coiling, the fiber would require almost 3 m to get such pump absorption and the fiber used in Figure 3a was only 107 cm long. With shorter fiber, the beneficial effect of twisting to the laser performance is pronounced. The slope efficiency of the hexagonal fiber under test is lower also due to higher background losses in this experimental fiber that were about 3 dB/m in the 2-µm spectral range. On the other hand, the Tm-doped panda fiber had background losses less than 0.2 dB/m and its length was closer to the optimum fiber length, yet still the beneficial effect of coiling and twisting was apparent also in the case of the laser experiment with the panda fiber.

As we have proved the advantages of the fiber twist both theoretically and experimentally, we have fabricated Tm-doped fiber with the twist frozen in the drawn fiber by introducing spinning of the preform during the fiber drawing process. The fiber was fabricated by the nanoparticle doping and shaped using the CO_2_ laser shaping discussed above. The inner cladding cross section was shaped in the form of octagon rather than hexagon in order to facilitate splicing with round fibers. The fiber had 3 wt % concentration of Tm and it was drawn in four different samples: (1) fiber with circular inner cladding of 125 µm and core of 19 µm diameter, and three samples with octagonal inner cladding of 120 µm flat-to-flat dimension and core of 20 µm diameter, the three samples differ in terms of preform spinning and corresponding twist rate: (2) 0 °/m, preform was not spun, (3) 0.8 °/mm, i.e., 2.24 rotation per meter, (4) 1.6 °/mm, i.e., 4.55 rotation per meter.

**Figure 4:**
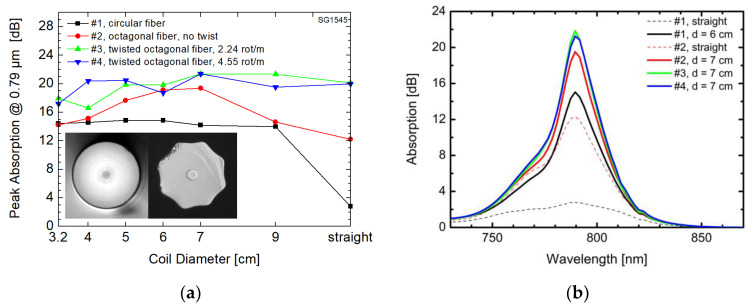
(**a**) Peak absorption versus coil diameter for four different fiber samples drawn from the same preform. Fiber cross sections are apparent from the microscope photographs in the inset; (**b**) Spectral absorption around the pump wavelength of 0.79 µm for selected coil diameters of the same four fiber samples.

Although the shape of the inner cladding cross section indeed affects the pump absorption, see for example, the study of inner claddings with periodic perturbation of their outer shapes [66], we showed that the effect of unconventional coiling on pump absorption is stronger than the effect of the inner cladding shape as long as its circular symmetry is broken. We measured separately the spectral absorption of a 2-m long pieces of all four fiber samples at around the wavelength 0.79 µm for straight condition as well as for different coiled conditions (the fiber was coiled on cylindrical spools with different radii). The peak absorptions for the four fiber samples are presented in Figure 4a where the lines between the experimental points are used for better representation of the trends only. Each point in Figure 4a represents one spectral absorption measurement for the given fiber sample under certain coiling condition. Some of the measured absorption spectra (corresponding e.g., to straight or to coiled fibers on cylinder with optimal diameter reaching peak absorption values) are compiled together in Figure 4b for better illustration. The same colors of the lines are used in both Figure 4a,b to represent those four different fiber samples. The twisting effect on the peak absorption could be seen in Figure 4a for straight octagonal fibers where the peak absorption of 12 dB for untwisted fiber (red color) was increased to 20 dB by twisting (green and blue colors). The pump absorption can be further improved for untwisted fiber (red color) by its coiling and there is apparently a local maximum of absorption for the coil diameter of about 7 cm. It is in accordance with pump field squeezing and decentering effects theoretically predicted by numerical models [62,63]. The circular fiber (black color) had poor peak absorption of less than 3 dB for straight condition, as expected, and it can be improved to about 14 dB by coiling the fiber.

It is apparent from the above discussion, one can conclude that the fiber twist improves the pump absorption in noncircular DC fibers even in the case of straight or coiled fibers with large coil radii. This new concepts including preform spinning shall result in highly efficient lasers of a small footprint and reduced need for cooling that shall have a great potential for applications where low power consumption, tightly limited space, and low weight are required.

## 4. Fused Fiber Components for 2-Micrometer Spectral Range

**Figure 5:**
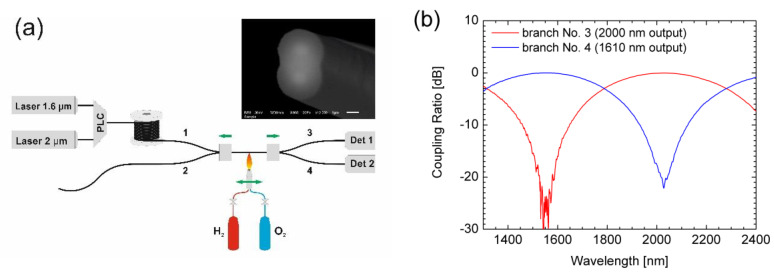
(**a**) Setup of the tapering rig for producing fiber components by the fused-biconically-tapered fabrication method. PLC: Planar lightwave circuit; Det: detector. Inset: scanning-electron-microscope image of the cross section near the waist position of the tapered region (the waist width and height as orientated in the shown inset were 3.7 μm and 5.5 μm, respectively); (**b**) Spectral transmission of the wavelength division multiplexers (WDMs) for combination of the pump and the signal in Tm fiber lasers.

Together with the SQS Fiber Optics company (Nova Paka, Czech Republic), we have developed a number of passive fiber components for fiber lasers operating at the 2-μm spectral range. A tapering rig, which is schematically shown in Figure 5, was used for this purpose. The biconical tapering-fusion process was realized by hydrogen-oxygen flame. In this way, fiber couplers and wavelength division multiplexers (WDMs) for combination of 1.6- and 2-μm radiation were produced. 

The components were produced using three commercially available fibers. The components’ characteristics were measured and compared as well as some limitations like the two-mode operation and the high bend loss were discussed [67].

A novel mode-field adapter for the tapered fused-fiber-bundle pump and signal combiners used in high-power DC fiber lasers was reported in [68]. The adapter allowed us to optimize the signal-mode-field matching on the input and output fibers whilst the combiner signal branch losses are significantly reduced. We demonstrated the mode-field adapter optimization procedure on a combiner based on commercially available optical fibers where 1.55- and 2-μm signal wavelengths were considered.

## 5. Diffractive Optics

Transmission properties of optical fibers can be modified by micro-patterning of the fiber facet. Different techniques for the fabrication of micro-optical elements directly on cleaved or polished end fiber facets could be employed. They are based on milling, etching, or additive manufacturing (focused ion-beam milling, electron-beam lithography, photolithography, interference lithography, two-photon polymerization 3D printing, nanoimprint technology, laser micromachining). Micro-patterning techniques of milling or etching the microstructures directly on the optical fiber facet attracted great interest for high-power laser applications. We have investigated leaky-mode resonant gratings (resonant metallo-dielectric gratings) used as a wavelength- and polarization-selective element in fiber lasers [69]. Resonant leaky-mode diffraction gratings are phase-matching structures with zero-order diffraction efficiency that could be close to 100% in reflection. They allow to simultaneously excite and extract a guided mode of the adjacent waveguide. We have fabricated a sub-wavelength diffraction grating by focused ion beam milling in a high-refractive-index layer deposited on the perpendicularly cleaved facet of a large mode area fiber [70]. The diffraction grating was tested in a Tm-doped fiber laser. It had different reflectivities for TE and TM polarized light and was used as a low reflectivity output mirror integrated with an intracavity polarizer. The laser with diffraction grating at the fiber end demonstrated slightly increased threshold power with almost the same slope efficiency compared to a laser with perpendicularly cleaved output fiber.

Another diffractive element widely used as mirror in fiber lasers is the fiber Bragg grating (FBG). Typically, a piece of fiber with an inscribed FBG has to be spliced to the active optical fiber during the laser cavity fabrication. Splices always introduce losses to the fiber system but they could eventually generate laser resonator failure if they are placed inside the cavity. A monolithic Tm-doped fiber laser with a pair of fiber Bragg gratings (FBGs) written into Tm-doped fiber using a two-beam interferometry and a deep ultraviolet (DUV) femtosecond laser source was reported in ref. [71]. To the best of our knowledge, this was the first published RE-doped fiber laser with a FBG pair written by DUV femtosecond laser radiation. The monolithic architecture allowed a simple setup of the laser made of a single fiber only with the benefit of robust and reliable all-fiber configuration. Correlation between the pump power and the laser wavelength was presented. Finally, the laser characteristics were compared to a fiber laser with FBGs inscribed into passive fibers that were spliced to the active Tm-doped fiber.

A monolithic fiber laser with a pair of FBGs which were inscribed directly into an erbium/ytterbium co-doped fiber using a direct-write, plane-by-plane femtosecond laser inscription were reported in ref. [72]. This inscription technique allowed complete spatial control of the refractive index change in the fiber core and eliminated fiber splices since no passive fibers with FBGs were needed. The gratings were characterized in transmission and reflection. The performance of the fiber laser was compared for fiber coiled into a circular or into a kidney shape. The kidney shape of the fiber coil improved the laser slope efficiency against the circular shape.

## 6. Applications in Mode-Locked Fiber Lasers

Mode-locked fiber lasers operating at 2-μm spectral range have been attracting increasing interest in the last decade as light sources for many industrial or medical applications [73]. Passively mode-locked fiber lasers have a relatively simple design. They are typically used as sources of high-quality ultrashort (sub-ps) pulses where intracavity saturable absorbers are used to carve the pulse profile.

A compact all-fiber ring laser which was passively mode-locked by using graphene-based saturable absorber was demonstrated in [26]. In this laser, a Tm-doped silica-based optical fiber with increased fluorescence lifetime of the ^3^F_4_ level, achieved by modification of the local environment of Tm ions by high content of alumina, was used. In cooperation with colleagues from Wroclaw University of Technology, a Ho-doped mode-locked fiber laser in all-fiber configuration using a graphene saturable absorber was demonstrated [74]. 

Artificial saturable absorbers–schemes that exploit optical Kerr effect in glass fibers, including nonlinear polarization rotation, have advantages in the principally wavelength insensitive operation and the fast response on femtosecond timescales. The laser system could be compact when the nonlinear polarization rotation scheme is used since it requires only a short piece of fiber. Elimination of bulk polarizers in the laser system, e.g., by using fiber gratings, results in an all-fiber configuration that makes the system even more robust.

An all-fiber passively mode-locked Tm-doped fiber laser which used an in-line fiber polarizer made of 45°-plane-by-plane-tilted fiber grating inscribed by a femtosecond laser was demonstrated in ref. [75]. The grating had a high contrast and a broad operating wavelength. It was combined with a polarization controller to operate as an artificial saturable absorber through nonlinear polarization rotation. Inscription of tilted fiber gratings by the plane-by-plane direct writing technique with a femtosecond laser [76], offers additional flexibility of the grating design compared to the classical grating inscription using UV light and phase masks.

## 7. Applications in Broadband Light Sources at Around 2-μm Wavelengths

Wideband fiber amplified spontaneous emission (ASE) sources are expected to provide spectral power density of several orders of magnitude higher than halogen bulb sources coupled into a single-mode fiber. ASE sources working at 2-μm wavelengths are usually based on Tm- and Ho-doped fibers. Particularly, large-mode area DC fibers are suitable for high-power wideband ASE sources. Experimental demonstration of two extremely wideband ASE sources is presented in [77] where a high bandwidth was achieved by combination of the forward and the backward ASEs generated in a core pumped Tm:Ho co-doped fiber. A pair of wideband fused fiber couplers was used in the set-up. The first ASE source with an integrated power of approximately 1.3 μW was spectrally flat with a −10 dB bandwidth of 645 nm. The second ASE source was optimized for spectroscopy having an integrated power of 9 μW and covering a spectral band of more than 800 nm. Two methods of increasing the bandwidth of Tm-doped fibers-based ASE sources were presented in ref. [78].

A broadband Tm-doped fiber ASE source working around the 1.85 µm spectral range was reported in ref. [79]. It was, to the best of our knowledge, the broadest ASE source to date based on core-pumped Tm-doped fiber without internal spectral filtering and with high output power. It has an output power exceeding 90 mW and a full width at half-maximum of the spectrum greater than 155 nm.

Self-swept fiber lasers represent a unique case of longitudinal-mode-instability lasers. We have reported self-sweeping of the laser wavelength in a Ho-doped fiber laser, which was, to our knowledge, the first observation of self-swept fiber laser beyond 2000 nm [80]. The sweeping was observed with rate ~0.7 nm/s from longer towards shorter wavelengths in the ~4 nm interval. The model published in [81] is an important step towards the understanding of origins of the self-sweeping of fiber lasers.

## 8. Demonstration of Material Processing by All-Fiber Tm-Laser Operating at 2 Microns

**Figure 6:**
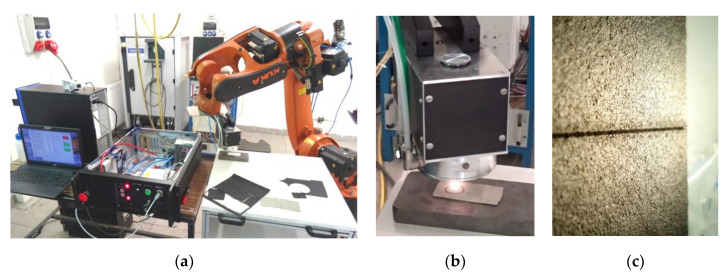
(**a**) Thulium all-fiber laser material processing testing workplace with robotic arm; (**b**) Detail of the laser head during cutting of synthetic leather with thickness of 1 mm; (**c**) Detail of a cut in SBR (Styrene Butadiene) rubber material.

As a result of a joint project with UCT Prague and industrial partners SQS Fiber Optics and Matex PM companies, a Tm all-fiber laser intended for material processing was developed. Brief experiments were performed on cutting and engraving a variety of materials like plastics (acrylic glass PMMA, polystyrene, polyurethane), NBR rubber, synthetic leather, paper, and steel. All tests on these different materials were performed under the same conditions (laser output power 20 W, robotic arm speed 10 mm/s, focal distance 25 mm) just as a demonstration of the in-house-developed laser and laser head. The laser workplace with robotic arm and some details are shown in Figure 6.

Although the process parameters of the laser were not optimized, it was demonstrated that the laser could be involved in material processing where, e.g., CO_2_ lasers are widely used nowadays. The benefits of the tested Tm fiber laser against other types of lasers are in general discussed in [7] and [8] and could be at least the more “eye-safer” 2-µm wavelength, the stronger absorption bands of some materials in this spectral region, and the all-fiber design of the laser (compact and robust device that could be situated in clean environment far away from the processing workplace-the laser light could be delivered by a processing optical fiber, e.g., to a robotic arm or even simultaneously distributed to more processing workplaces).

The project was focused on the development of the laser, some of the laser components and the laser head, but it was not aimed on the material processing itself. Therefore, further improvement of the laser system and optimization of the processing parameters were deferred and to be carried in the near future.

## 9. Conclusions

New results on the investigation of the twisting effects to the pump characteristics of double-clad optical fibers were presented for the first time in this paper to supplement the brief review of our recent progress in the research of thulium- and holmium-doped optical fibers and fiber components, fiber lasers, and related topics primarily dedicated to the 2-µm spectral range applications. Nanoparticle doping method of modification of the local environment of thulium ions by high alumina nanoparticle co-doping proved to increase the thulium quantum conversion efficiency important for fiber laser applications. Coiling and twisting of double-clad fibers leads to substantial increase of the pump absorption along the fiber as we have demonstrated on fibers with hexagonal and octagonal shape of the inner cladding. A thermal-based preform shaping by CO_2_ laser was employed prior to drawing fibers which enhanced their pump absorption efficiency. The paper referred to the development of several passive components and diffraction gratings nanostructured on the fiber end, all intended for fiber lasers operated around 2 µm. Finally, mode-locked fiber lasers, a monolithic fiber laser with a pair of FBGs inscribed directly into the erbium/ytterbium co-doped fiber, as well as broadband light sources and self-sweeping of fiber lasers were mentioned to highlight our research activities in the field of fiber devices working at around 2 μm. Experiments to demonstrate the material processing capabilities of in-house-made thulium all-fiber laser operating at 2 microns were presented despite further improvement of the laser system and optimization of the processing parameters is expected to be carried out in the near future.
